# The relationship between mosquito abundance and rice field density in the Republic of Korea

**DOI:** 10.1186/1476-072X-9-32

**Published:** 2010-06-23

**Authors:** Erin E Richards, Penny Masuoka, David Brett-Major, Matthew Smith, Terry A Klein, Heung Chul Kim, Assaf Anyamba, John Grieco

**Affiliations:** 1 Department of Preventive Medicine and Biometrics, Uniformed Services University, Bethesda, Maryland, USA; 2 Military Tropical Medicine Course, Navy Medicine Manpower Personnel Training and Education Command, Uniformed Services University, Bethesda, Maryland, USA; 3 Biospheric Sciences Branch, Goddard Space Flight Center, National Aeronautics and Space Administration, Greenbelt, Maryland, USA; 4 Force Health Protection and Preventive Medicine, 65th Medical Brigade/MEDDAC-Korea, Seoul, Republic of Korea; 5 5th Medical Detachment, 168th Medical Battalion, 65th Medical Brigade, Seoul, Republic of Korea

## Abstract

**Background:**

Japanese encephalitis virus (JEV), the causative agent of Japanese encephalitis (JE), is endemic to the Republic of Korea (ROK) where unvaccinated United States (U.S.) military Service members, civilians and family members are stationed. The primary vector of the JEV in the ROK is *Culex tritaeniorhynchus*. The ecological relationship between *Culex *spp. and rice fields has been studied extensively; rice fields have been shown to increase the prevalence of *Cx. tritaeniorhynchus*. This research was conducted to determine if the quantification of rice field land cover surrounding U.S. military installations in the ROK should be used as a parameter in a larger risk model that predicts the abundance of *Cx. tritaeniorhynchus *populations.

Mosquito data from the U.S. Forces Korea (USFK) mosquito surveillance program were used in this project. The average number of female *Cx. tritaeniorhynchus *collected per trap night for the months of August and September, 2002-2008, was calculated. Rice fields were manually digitized inside 1.5 km buffer zones surrounding U.S. military installations on high-resolution satellite images, and the proportion of rice fields was calculated for each buffer zone.

**Results:**

Mosquito data collected from seventeen sample sites were analyzed for an association with the proportion of rice field land cover. Results demonstrated that the linear relationship between the proportion of rice fields and mosquito abundance was statistically significant (R^2 ^= 0.62, r = .79, F = 22.72, p < 0.001).

**Conclusions:**

The analysis presented shows a statistically significant linear relationship between the two parameters, proportion of rice field land cover and log_10 _of the average number of *Cx. tritaeniorhynchus *collected per trap night. The findings confirm that agricultural land cover should be included in future studies to develop JE risk prediction models for non-indigenous personnel living at military installations in the ROK.

## Background

**Figure 1:**
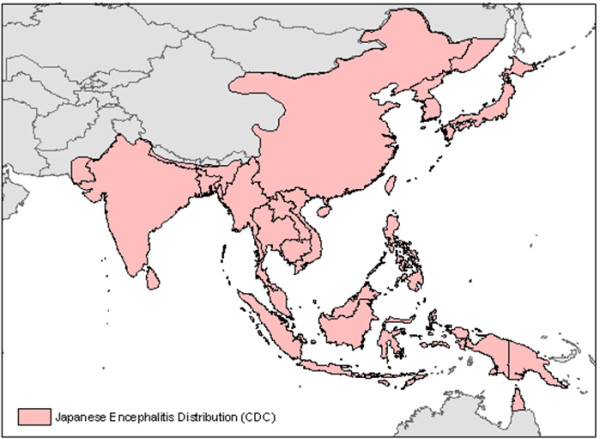
**Geographical distribution of Japanese encephalitis**. Geographical distribution of Japanese Encephalitis as published in *CDC Health Information for International Travel 2010 *[13]. Guam and Saipan (not shown on this map) have also had cases of Japanese Encephalitis.

Japanese encephalitis virus has been identified by the U.S. National Center for Medical Intelligence as operationally important to the U.S. military. Similarly, the pathogen is important to unvaccinated personnel from other militaries around the globe, expatriates, and travelers [1]. JE is a potentially debilitating and deadly flavivirus that is endemic in rural areas in east, south, and southwest Asia. Successful vaccination programs have diminished the number of illnesses in many countries, causing many to consider the disease to be rare and exotic [2]. However, the JEV is maintained in populations of wading birds and its primary vector *Culex tritaeniorhynchus *is plentiful in many Asian countries. It has been shown that the number of JEV vector mosquitoes collected in an area is highly correlated with the number of JE patients in that area [3]. While a reliable JE vaccination exists, this disease still affects many Southeast Asian countries [4,5]. Recent outbreaks in the last five years (2005 through 2010) have occurred in the indigenous population and unvaccinated expatriates and travellers in India, Nepal, Taiwan, Malaysia, China, and Vietnam [6-12]. Recent maps showing the geographical distribution of JE have been published in several different papers [2,12,13]. The JE distribution map in Figure 1 is based on the U.S. Centers for Disease Control and Prevention (CDC) 2009 version of the map currently distributed to international travellers [13].

While JE clinical manifestations occur only in a small number that become infected, the case fatality rate can be as high as 40% for those demonstrating symptoms. Permanent neurologic sequelae are observed in 40 to 50% of those who survive severe disease, resulting in minor to severe complications and early death [14,15]. Furthermore, JE is often misdiagnosed and lacks effective treatment beyond supportive care [16,17].

U.S. military personnel have been stationed in the ROK since World War II. Currently, programmed extended tour lengths have greatly increased the number of accompanying family, placing family members and young unvaccinated children at risk of infection. While an established Korean pediatric JEV vaccination program has greatly reduced the number of cases occurring in the Korean population, JEV continues to be of military public health concern in the ROK because U.S. personnel are not vaccinated for JE. Because of this disparity, human disease rates in the indigenous population are unreliable for estimating transmission and disease risks to U.S. military, civilian, and family members stationed there.

*Culex tritaeniorhynchus *is exophagic, prefers zoonotic and domestic hosts, and tends to feed interspecifically [18]. Primary vertebrate reservoirs for JEV are large water birds of the family *Ardeidae*, while wild and domestic pigs are amplifying hosts [17,19]. *Cx. tritaeniorhynchus *is highly susceptible to viral infection; studies suggest that over 30% become infected after feeding on a viremic pig [20]. Mosquito populations are dependent on environmental and seasonal factors for their propagation. In the ROK, seasonal occurrence of *Cx. tritaeniorhynchus *are reported between mid-June and early October, with adult peak populations from late July through early September [21-26]. JEV infections within vector populations peak in late July and early August, with human disease rates peaking in late August and September [15,27]. *Culex tritaeniorhynchus *breed in open sunlit temporary and permanent water habitats with vegetation, and have an average flight range of 1.5 km [4,28]. The relative abundance of *Cx. tritaeniorhynchus *is closely related to local rice agricultural practices, where wetland rice fields and similar irrigation systems provide an optimal habitat for larval development [18]. The ecological relationship between mosquitoes and rice fields has been studied extensively [29-31]; results show that these man-made breeding sites result in a greater prevalence of *Cx. tritaeniorhynchus *than natural breeding sites [32,33].

Remote sensing and Geographical Information Systems (GIS) are increasingly used in disease epidemiology to map vector and disease distributions so that vector control efforts and disease intervention strategies are administered efficiently and effectively [29]. Past research has demonstrated that remote sensing and GIS capabilities can be used successfully to identify breeding habitats of *Cx. tritaeniorhynchus *in Japan, the ROK, India, and Australia [18].

Past studies on relative seasonal abundance and geographical distribution of *Cx. tritaeniorhynchus *in the ROK have contributed to the success and development of a JE disease surveillance systems for local populations [34]. These surveillance systems have been based on monitoring vector populations and seroprevalence in swine from selected slaughterhouses; while such surveillance systems have been effective, they have a negative impact on the limited human resources and man-power available to public health. For this reason, the Uniformed Services University, the Armed Forces Health Surveillance Center, Global Emerging Infections Surveillance and Response System (AFHSC-GEIS), the National Aeronautics and Space Administration (NASA) Goddard Space Flight Center (GSFC), and the 65^th ^Medical Brigade-Korea, began working together to develop a JEV risk model in the ROK that incorporates remote sensing, land cover, elevation, and historical climate data.

The goal of this project was to determine if quantification of wetland rice field land cover via remote sensing and GIS techniques could feasibly be used as a parameter in a larger risk model to predict the abundance of *Cx. tritaeniorhynchus *at U.S. military installations in the ROK. While past studies have shown a correlation between *Cx. tritaeniorhynchus *collected at cow and pig sheds and surrounding rice field acreage [35,36], this study examines the number of Cx. tritaeniorhynchus collected on primarily urban military bases and compares the number of mosquitoes collected to the amount of rice field surrounding those bases. Quantifying the relationship between the amount of rice fields surrounding U.S. military installations in the ROK and the abundance of *Cx. tritaeniorhynchus *captured at these sites will enhance the U.S. military's capability to predict the risk of JEV transmission to military, civilians, and family members assigned to the ROK. This proposed risk model would be used in conjunction with a predictive model for seasonal JEV transmission using real-time data and would serve as a disease risk monitoring system for U.S. military and civilians living in the ROK. The development of an accurate risk model based on remotely sensed land and climate data will allow for more efficient detection of increased JE risk to U.S. military populations in the ROK. Additionally, the risk model would diminish reliance on man-power intensive surveillance systems, and may provide rationale for the implementation of mandatory JEV vaccination to USFK military, civilian, and family members assigned to the ROK.

## Methods

### Study Area

**Figure 2:**
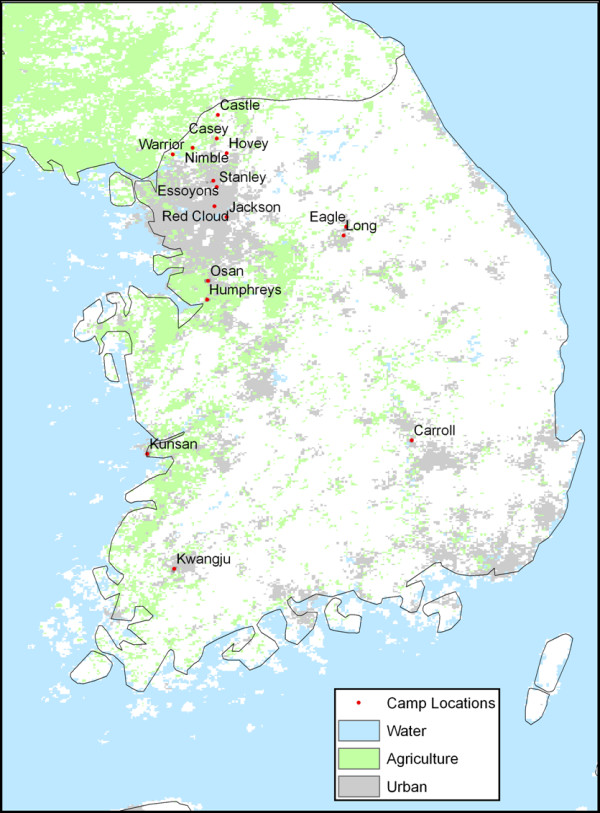
**Study area**. The following military installations within South Korea are shown: Camp Humphreys, Camp Carroll, Gwangju Air Base (Kwangju), Warrior Base, Camp Red Cloud, Camp Stanley, Camp Jackson, Camp Essoyons, Camp Long, Camp Eagle, Camp Casey, Camp Castle, Camp Nimble, Camp Hovey, Osan Air Base, and Gunsan Air Base (Kunsan). Land cover obtained from MODIS Land Cover Group http://www-modis.bu.edu/landcover.

The study area comprised seventeen U.S. military installations in the ROK and their immediate surrounding areas. Military installations included in this study are a convenience sample based on availability of high resolution images and mosquito data provided by the USFK mosquito surveillance program. Installations included in this study are Camp Humphreys, Camp Carroll, Gwangju Air Base, Warrior Base, Camp Red Cloud, Camp Stanley, Camp Jackson, Camp Essoyons, Camp Long, Camp Eagle, Camp Casey, Camp Castle, Camp Nimble, Camp Hovey, Osan Air Base, and Gunsan Air Base (Figure 2).

Reorganization of the U.S. military in the ROK has led to the realignment of existing military installations and closure of others. Camp Humphreys, which has been under construction since late 2007, is one of the largest installations and has been augmented in order to accommodate an additional 20,000 relocated military, civilian, and family members. Wetland rice fields adjacent to Camp Humphreys were purchased for installation expansion, with fill dirt used to elevate the low-lying rice paddies for development, thus changing the local landscape. A portion of the secured rice paddies are programmed to lay fallow, thereby contributing to a possible increase in *Cx. tritaeniorhynchus *populations during the wet Monsoons (June-September). Two images of Camp Humphreys were used in this project; one image is a 2006 QuickBird image and the other is a 2008 IKONOS image of the same location. The rice field density statistics used in analysis for Camp Humphreys is based on the 2006 image only. The 2008 image shows the amount of rice fields lost as a result of installation construction. The two images were used in an exploratory fashion to help describe how changes in local land cover effect the abundance of *Cx. tritaeniorhynchus*.

### Mosquito Data

Retrospective mosquito data were provided by the USFK, which conducts routine mosquito surveillance on U.S. military installations to monitor all mosquito species. Mosquitoes were collected weekly using un-baited New Jersey (NJ) light traps in accordance with USFK preventive medicine protocol from May through October, 2002 to 2008. One to eight traps were established at each military installation. Collections were sent to the Entomology Section, 5^th ^Medical Detachment, for identification where data on adult female mosquitoes (i.e. species, trap night, month, and year) were prepared. A summary of the total number of mosquitoes (males plus females) collected at each location from 2002 to 2008 is listed in Table 1. Although NJ light traps are relatively inefficient for collecting mosquitoes when compared to newer technologies, they are easy to operate and data can be compared with collections made over more than 20 years of surveillance in the ROK.

**Table 1:** Number of *Culex tritaeniorhynchus *mosquitoes collected in the ROK from 2002 to 2008 by month*

	MAY	JUN	JUL	AUG	SEP	OCT	Total
**2002**	1	28	3724	27515	17995	524	49787
**2003**	0	7	315	1790	2282	38	4432
**2004**	0	12	650	32957	9446	1487	44552
**2005**	0	2	294	4264	2502	136	7198
**2006**	0	6	40	580	1046	6	1678
**2007**	1	12	986	2976	845	122	4942
**2008**	0	5	584	2421	1584	33	4627

*Data are not adjusted for number of trap nights. The numbers in this table include both male and female mosquitoes and those trapped at Gunsan, which was later dropped from the study.

The final mosquito count at each military installation used in the main analysis of this study is an average from August to September from 2002 to 2008. August and September were selected for analysis to capture the peak *Cx. tritaeniorhynchus *breeding cycle [21]. To reduce the effects of sampling variation between years and installations, data were standardized as the number of female mosquitoes collected per trap night per month [mosquitoes per trap night = total female mosquitoes collected/(number of traps * number of nights)]. The final mosquito capture rate for each military installation was calculated by adding the number of mosquitoes per trap night for months August and September from 2002 to 2008, then dividing by the number of nights mosquito sampling was conducted during that time period.

### High Resolution Satellite Data

One IKONOS and ten QuickBird high resolution images, encompassing visible and near infrared data, taken between 2004 and 2008 were used for land cover classification. QuickBird is a commercial earth observation satellite operated by DigitalGlobe http://www.digitalglobe.com that records multispectral imagery at 2.6 meter resolution at nadir. IKONOS is a similar commercial earth observation satellite operated by GeoEye http://www.geoeye.com that provides multispectral images at four meter resolution. Although the QuickBird images have a higher resolution than the IKONOS image, comparison studies have demonstrated that land classification outcomes are analogous for both image types [37]. Within each of the ten QuickBird images and one IKONOS image, military installations were located using latitude and longitude coordinates. Processing of image data involved two steps: digitizing and area computation.

Rice fields were identified using photointerpretation and manual digitization via ArcGIS^® ^9.3 software (Environmental Systems Research Institute, Redlands, CA, USA, http://www.esri.com). The border of each military installation was digitized using publically available installation maps as aids. Buffers were used to designate the study inclusion area. The buffer area surrounding each military installation was based on the average flight range of *Cx. tritaeniorhynchus *[4], and was set at 1.5 km from the digitized military installation border. Rice fields within the buffer area of each military installation were then manually digitized as polygons. Manual digitization was chosen because the majority of images available for this project were acquired during the winter months when rice fields were bare and spectrally similar to other land cover, making automated classification difficult. Rice field land cover was digitized as large polygons consisting of many individual rice fields. Other non-rice features such as irrigation ditches and small roads may have been included in the rice field land cover. To minimize variation, all digitization was completed by one researcher in a consistent manner.

**Figure 3:**
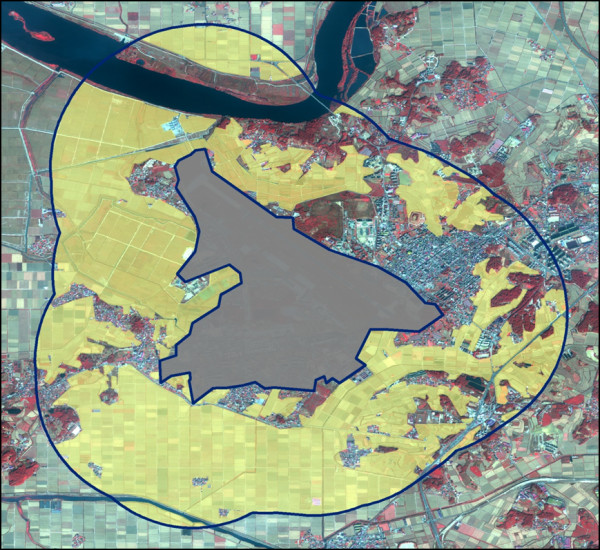
**Digitized rice field data example**. The above is an image of a military installation located in the ROK taken in October. The base is shown with a solid grey cover; the extent of the buffer zone area is depicted with a dark blue line; rice fields are shown with a solid yellow cover. The buffer zone area extends 1.5 km from the military installation perimeter.

To obtain area measurements in kilometers, the digitized military installations buffer area and rice field shape files were reprojected from a Geographic Coordinate System (GCS) to a Universal Transverse Mercator (UTM) map projection. The buffer area at each military installation was calculated and reported in square kilometers. The digitized rice field polygons within the buffer area of each military installation was calculated and reported in km^2^. To account for variation in installation size and thus buffer area size, rice field densities were calculated as the proportion of rice fields within the buffer area at each installation (rice fields proportion = rice field area/military installation buffer area). An example of digitized rice field data is presented in Figure 3.

### Rice Field Analysis

Simple linear regression analysis, using the SPSS^® ^statistical program (SPSS Inc., Chicago, IL, USA) was conducted on the data using two continuous variables, rice field density and log_10 _of the average number of *Cx. tritaeniorhynchus *per trap night from 2002-2008. The rice field density statistic representing the proportion of rice fields surrounding Camp Humphreys was based on the 2006 image only.

A regression model was developed from the data to predict mosquito abundance at Camp Humphreys after environmental changes in 2007 altered the amount of rice fields surrounding the installation. Rice field density in the 2008 Camp Humphreys image was applied to the regression model to determine a predicted mosquito count and then compared to the observed 2008 number of mosquitoes per trap night. This test was used to explore the model's ability to predict the abundance of female *Cx. tritaeniorhynchus *collected at an installation.

Mosquito prevalence trend analysis showed extreme variation over time. Years 2002 and 2004 had abnormally high counts of *Cx. tritaeniorhynchus *when compared to all other years (Table 1). An observational retrospective study of mosquito prevalence at U.S. military installations in the ROK found that *Cx. tritaeniorhynchus *was the most common mosquito species collected in 2002 [38]. In 2002, due to a high number of mosquitoes collected at Gunsan Air Base, the overall average of *Cx. tritaeniorhynchus *populations in the ROK increased by 27.2%, while populations of other mosquito species remained relatively constant during the same time period [38]. The increased number of *Cx. tritaeniorhynchus *caught at Gunsan Air Base in 2002 and 2004 could be due to the use of more efficient collection methods during these two years. At Gunsan, NJ light traps were baited with dry ice during collections in 2002 and the Mosquito Magnet^® ^was used for collections in 2004 [38]. The Mosquito Magnet is the most effective trap for collecting *Cx. tritaeniorhynchus*; capturing up to 650 times as many mosquitoes as the NJ light trap [23]. Primarily because trapping methods were substantially different at Gunsan than those at all other installations in the study sample, Gunsan Air Base was excluded from the final analysis.

*A priori *statistical power analysis stated that a sample of sixteen sites carried 80% power to detect the proportion of variability between mosquito abundance and rice field density in the model with a coefficient of determination (R^2^) of 0.65. For our purposes, an R^2 ^of 0.65 was desired. The correlation coefficient (r) was also used in this analysis to elucidate the relationship between mosquito abundance and the rice field density seen in the data.

## Results

**Figure 4:**
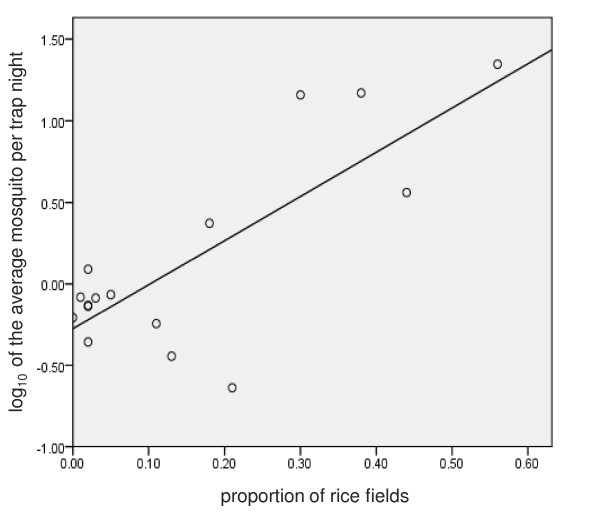
**Fitted curve of model**. The above is a scatter plot of the data and the resulting regression line (y = 2.71 × -0.28). Each dot represents data from one installation.

Average mosquito counts per trap night and the corresponding rice field density statistics are presented for each installation in Table 2. The area surrounding half of the installations in this study were comprised of less than 10% rice fields. This is in sharp contrast to installations that were surrounded by the most rice fields; coverage ranged between 30-56% of the land surrounding five installations. Mosquito data were log_10 _transformed prior to regression analysis. Analysis conducted using log_10 _transformed mosquito data from 2002-2008 demonstrated a statistically significant linear relationship between rice field density and mosquito abundance (R^2 ^= 0.62, r = .79, F = 22.72, p < 0.001). A scatter plot of the data with the regression line is presented in Figure 4.

**Table 2:** Rice field density and average number of mosquitoes collected per trap night, by installation**

Installation	Proportion of Rice Fields in Buffer Area	Monthly Average Number Female Mosquitoes per Trap Night 2002-2008 (Aug-Sept)
Camp Jackson	0.00	0.62
Camp Casey	0.01	0.83
Camp Hovey	0.02	0.74
Camp Essoyons	0.02	0.73
Camp Nimble	0.02	0.44
Camp Mobile	0.02	1.23
Camp Red Cloud	0.03	0.82
Camp Castle	0.05	0.86
Camp Stanley	0.11	0.57
Camp Long	0.13	0.36
Camp Carroll	0.18	2.36
Camp Eagle	0.21	0.23
Gwangju Air Base	0.30	14.40
Warrior Base	0.38	14.81
Osan Air Base	0.44	3.64
Gunsan AB	0.53	222.42
Camp Humphreys	0.56	22.20

**Gunsan was dropped from subsequent analysis.

The resulting linear model [y = 2.71 × -0.28; where y equals the log_10 _of the average mosquitoes per trap night, and × equals the proportion of rice field land cover] was used to predict the abundance of post-construction *Cx. tritaeniorhynchus *at Camp Humphreys using the 2008 image. The data reveals that for every 10% increase of rice field land cover surrounding an installation, an 86% increase of female *Cx. tritaeniorhynchus *per trap night can be expected.

In an exploratory analysis the regression model was used to predict the abundance of *Cx. tritaeniorhynchus *at Camp Humphreys in 2008. Despite major changes to the Camp Humphreys area, the proportion of rice fields surrounding the installation increased by only 1%. The number of predicted mosquitoes per trap night was 18.2 for the 2008 Camp Humphreys image; the number of observed mosquitoes per trap night was 22.2.

## Discussion

This research addresses the issues of mosquito abundance and spatial patterns of agricultural land use. Emphasis has been put on determining the relationship between average *Cx. tritaeniorhynchus *collected per trap night and density of rice fields surrounding U.S. military installations in the ROK; as well as assessing the value of that relationship for inclusion in a larger disease risk model. The future application of rice field density as a parameter in models used to predict JEV disease risks can be based on the strength of linear association, as it is presented here.

Results revealed that 62% of the variation in the log_10 _of *Cx. tritaeniorhynchus *collected per trap night on base was accounted for by the density of rice fields in the area surrounding the military base. The remaining 38% can be explained by inherent variability or other variables, such as species breeding cycle, proximity to blood source, or regional climatic conditions [17,19-21,31]. Our data reaffirms past JE and *Cx. tritaeniorhynchus *research and suggests that rice fields account for a large portion of the mosquito's abundance but that populations cannot be predicted by rice fields alone [29-31,35,36].

A statistically significant linear association was demonstrated between rice field density and mosquito population. Given the strong relationship demonstrated between rice field density and mosquito populations, this variable should be considered when modelling *Cx. tritaeniorhynchus *propagation or JEV transmission risks.

Specific limitations to this study with regard to image data include the inability to automatically extract rice fields due to the lack of ground vegetation in many of the images used. Due to cloud cover and other limiting factors, the remotely sensed imagery used in analysis were not all acquired during the rice growing season, making automated land cover extraction software unable to accurately identify rice fields. While rice fields were extracted manually, the potential for misclassification of land cover remained. Field shape, image interpretation, and area photographs guided rice field identification. This study was also limited by the lack of images at each installation for each year or month within the study period. Thus, the authors were unable to explore variation in the correlation between rice field density and *Cx. tritaeniorhynchus *throughout time. This study, and potentially all studies conducted on military bases in the ROK, will be limited by the number of installations and number of installations within the USFK mosquito surveillance program. This limitation restricts analysis and does not allow the geographical variability among installations to be explored.

Despite limitations, this study has a number of strengths. It utilized USFK mosquito surveillance data collected consistently for over 8 years in the ROK. This data continues to be collected and can be similarly used in the future. It also uses high resolution remotely sensed imagery to determine rice field land cover. Using remotely sensed data to map rice field land cover is less time consuming than conventional ground survey methods used in the past to calculate agricultural land use.

## Conclusions

Using the data presented here, an overall statistically significant linear relationship between rice field density and *Cx. tritaeniorhynchus *abundance was found. Use of the regression model to predict mosquito abundance was inconsistent. Future studies to develop JE risk prediction models for non-indigenous personnel living at military installations in the ROK should include agriculture land use and irrigation patterns as variables.

There are a number of implications in this study for future research in JE risk prediction and *Cx. tritaeniorhynchus *population modelling in the vicinity of U.S. military installations in the ROK. First, automated risk models must utilize land cover maps derived from remotely sensed imagery acquired during the rice growing season. The use of images acquired with fallow rice fields could hinder the accuracy of automated rice field mapping, and could inhibit model efficiency. Second, if possible, imagery should be acquired for each month or year during the study period to facilitate the analysis of rice field density and *Cx. tritaeniorhynchus *within and among installations, and account for annual fluctuation in average mosquito per trap night.

## Abbreviations

AFHSC: Armed Forces Health Surveillance Center; CDC: Centers for Disease Control and Prevention; GCS: Geographic Coordinate System; GEIS: Department of Defense Global Emerging Infections Surveillance and Response System; GIS: Geographical Information Systems; GSFC: Goddard Space Flight Center; JE: Japanese encephalitis; JEV: Japanese encephalitis virus; NASA: National Aeronautics and Space Administration; NJ: New Jersey; ROK: Republic of Korea; USFK: United States Forces Korea; UTM: Universal Transverse Mercator.

## Competing interests

The authors declare that they have no competing interests.

## Authors' contributions

ER implemented the mapping project, analysis of the data, drafting of the manuscript, and contributed to the study design. PM conceived the study design, acquired land cover data and contributed to the coordination of the study, implementation of the mapping project, and revision of the manuscript. DB contributed to the study design and revision of the manuscript. MS participated in the creation of the mapping project and revision of the manuscript. AA contributed to the coordination of the study and advised on study design. HCK assisted with mosquito data collection, provided advice on mosquito data analysis and provided coordination with USFK. TAK collected mosquito data, provided advice on mosquito data analysis, provided coordination with USFK, and assisted with manuscript revision. JG provided project resources, advised on study design and analysis, and assisted in final editing. All authors have read and approved the final manuscript.
